# The *mcpC* mutant of *Salmonella enteritidis* exhibits attenuation and confers both immunogenicity and protective efficacy in mice

**DOI:** 10.3389/fmicb.2025.1548920

**Published:** 2025-02-10

**Authors:** Lu Zhang, Li Chen, Xuqiang Zhang, Yang Li, Qingfeng Zheng, Yun Li, Ning Li, Qiumei Shi, Yanying Zhang, Tonglei Wu

**Affiliations:** ^1^Hebei Key Laboratory of Preventive Veterinary Medicine, College of Animal Science and Technology, Hebei Normal University of Science and Technology, Qinghuangdao, China; ^2^Qinhuangdao Animal Husbandry Station, Qinghuangdao, China; ^3^Shijiazhuang Animal Products and Veterinary Drug Feed Quality Testing Center, Shijiazhuang, China; ^4^Tangshan Academy of Agricultural Sciences, Tangshan, China

**Keywords:** *Salmonella enteritidis*, *mcpC*, gene deletion, virulence, vaccine

## Abstract

**Background:**

*Salmonella enteritidis* (*SE*) is a Gram-negative, facultative anaerobic intracellular pathogen that not only causes disease and mortality in livestock and poultry but also contaminates animal-derived products, leading to foodborne illnesses in humans. This presents a significant threat to public health. To eliminate this pathogen, the development of novel vaccines targeting *SE* is imperative. Attenuated live vaccines are capable of eliciting robust immune protection against *SE*.

**Methods:**

In this study, an *mcpC* gene deletion strain (Δ*mcpC*) was constructed by the wild strain C50336, to evaluate its potential as a genetically engineered attenuated live vaccine. The virulence of Δ*mcpC* was assessed by examining its resistance to environmental stresses, biofilm formation capacity, motility, adhesion, invasion ability, intracellular survival, LD_50_, expression levels of virulence genes, and *in vivo* colonization ability. Furthermore, the immunogenicity of Δ*mcpC* was analyzed in mice by measuring specific IgG and SIgA antibody levels, lymphocyte proliferation, cytokine expression, and the protective efficacy of Δ*mcpC* vaccination.

**Results:**

Compared to the wild-type strain, Δ*mcpC* exhibited no significant changes in biofilm formation or adhesion to Caco-2 cells. However, Δ*mcpC* showed significantly reduced survival under acidic, alkaline, thermal, and oxidative stress conditions; markedly diminished motility; weakened invasion of Caco-2 cells; and reduced intracellular survival in RAW264.7 macrophages. The LD_50_ of Δ*mcpC* increased by 30-fold, and the expression levels of certain virulence genes were significantly downregulated. Additionally, Δ*mcpC* demonstrated significantly decreased colonization in the liver, spleen, and cecum of mice, indicating attenuated virulence. Immunization with Δ*mcpC* induced the production of specific IgG and SIgA antibodies, enhanced lymphocyte proliferation, upregulated cytokine expression, and achieved a 100% survival rate in immunized mice. These findings indicate that Δ*mcpC* provides effective immune protection in mice.

**Conclusion:**

This study demonstrates that deletion of the *mcpC* gene attenuates the virulence of *SE*. The Δ*mcpC* offers strong immune protection in mice, providing a solid foundation for the development of genetically engineered attenuated live vaccines against *SE*.

## Background

*Salmonella enteritidis* (*SE*) is a significant zoonotic pathogen that not only inflicts substantial economic losses on the livestock industry but has also emerged as one of the predominant serotypes causing salmonellosis in humans in recent years (Zhou et al., 2023; Chousalkar et al., 2018). While strict biosecurity measures can create *Salmonella*-free poultry farming environments, such measures are often economically unfeasible in many countries. Moreover, the widespread emergence of multidrug-resistant strains has raised concerns about the use of antibiotics. Vaccination remains a primary strategy for controlling *Salmonella* infections. Currently, inactivated vaccines, subunit vaccines, and live attenuated vaccines represent the major categories of *Salmonella* vaccines under development (Huberman et al., 2019).

Studies indicate that inactivated vaccines primarily induce humoral immunity and fail to elicit robust cell-mediated immune responses. As a result, booster immunizations are often required to achieve long-term protection (Deguchi et al., 2009). Subunit vaccines, while safe, typically require multiple doses to extend the duration of immunity and necessitate suitable adjuvants to trigger effective cellular immune responses. In contrast, live attenuated vaccines, when administered orally, adhere to the intestinal mucosa and mimic natural infection, thereby eliciting strong humoral and cell-mediated immune responses (Van Immerseel et al., 2005). The identification of virulence genes is crucial for developing gene-deletion-based live vaccines. This approach has been successfully employed in the development of various vaccines, including those for *Brucella* spp., *Salmonella* Typhimurium, *Yersinia pestis*, *Vibrio anguillarum*, and *Edwardsiella tarda* (Yang et al., 2015; Liu et al., 2018; Park et al., 2020; Cote et al., 2021; Zabalza-Baranguá et al., 2023).

*SE* must overcome multiple defense mechanisms inside and outside the gastrointestinal tract to establish infection in the host. Upon entering the digestive system, the first challenge it encounters is the bactericidal effect of gastric acid. Gastric acid, being a strongly acidic environment, inhibits or kills most microorganisms that reach the stomach. To survive, *SE* expresses high levels of acid shock proteins, such as ATPase, which help maintain intracellular pH balance and enhance its resistance to gastric acid. In the intestine, *SE* defends itself against bile and digestive enzymes by regulating its lipopolysaccharide (LPS) structure and producing anti-bile proteins, such as TolC. In the small intestine, it utilizes a type III secretion system (T3SS) to secrete effector molecules, including SipA, SipB, and SipC, which facilitate its invasion of intestinal epithelial cells. Within host cells, *Salmonella* forms a specialized membrane-bound compartment called the *Salmonella*-containing vacuole (SCV), which protects it from lysosomal degradation. After being phagocytosed by macrophages, *SE* injects T3SS effectors like SifA and SipC to inhibit the fusion of SCVs with lysosomes. Additionally, *Salmonella* can evade autophagy-mediated clearance by secreting effectors such as SseJ, allowing it to persist and proliferate within the SCV. In summary, the infection process of *SE* involves adapting to gastric acid, resisting bile, invading intestinal epithelium, evading the immune system, surviving intracellularly, and inducing inflammation. Each step relies on specific virulence factors and adaptive mechanisms, enabling *Salmonella* to establish infection in the gastrointestinal tract and beyond, leading to host disease. The loss of these virulence factors significantly attenuates the pathogen’s virulence. Consequently, researchers have utilized these virulence factors to develop various gene-deletion vaccines, achieving promising immune protection results.

Methyl-accepting chemotaxis protein C (McpC) is a methylation-based chemotaxis protein involved in bacterial chemotaxis, enabling directional movement in response to chemical gradients. By sensing and transmitting environmental chemical signals, McpC helps bacteria regulate their motility. This chemotactic ability is crucial for pathogenic bacteria to colonize and spread within the host, thereby playing a significant role in virulence. Additionally, McpC is involved in regulating biofilm formation, flagellar biosynthesis, and toxin production (Hickman et al., 2005; Berleman and Bauer, 2005; Harkey et al., 1994). As such, modulating McpC expression or function in pathogens may impact their infection efficiency and pathogenicity, making McpC a key target for studying bacterial virulence and infection mechanisms. Previous studies have demonstrated the role of methyl-accepting chemotaxis proteins (MCPs) in the pathogenicity of various bacteria, including *Pseudomonas aeruginosa*, *Campylobacter jejuni*, and *Vibrio cholerae* (Li et al., 2014; Sampedro et al., 2014; Nishiyama et al., 2016). Based on these findings, it is hypothesized that McpC contributes to the virulence of *SE*, a hypothesis that requires further experimental validation. By precisely deleting the *mcpC* gene, the pathogen’s virulence is significantly reduced while retaining its immunogenicity, thereby greatly enhancing safety and efficacy. Moreover, the vaccine strain produced by deleting the presumed virulence-associated gene *mcpC* can induce robust mucosal, cellular, and humoral immune responses, making it suitable for non-injection administration methods such as oral or spray vaccination, which facilitates large-scale application.

In this study, we constructed an *mcpC* gene deletion strain (Δ*mcpC*) using homologous recombination techniques and investigated its impact on *SE* virulence. The evaluation included analyses of stress resistance, biofilm formation, motility, adhesion, invasion, intracellular survival in macrophages, LD_50_, expression levels of virulence genes, and bacterial load in host organs. Additionally, we assessed the ability of Δ*mcpC* to induce immune responses and its protective efficacy in mice using an infection model. The findings provide a foundational basis for the development of genetically engineered live-attenuated vaccines targeting *SE*.

## Materials and methods

### Bacterial strains, cells and plasmids

Bacterial strains and plasmids used in this study are listed in Table 1. *Salmonella enteritidis* strain C50336, a wild-type strain, was preserved in the Key Laboratory of Preventive Veterinary Medicine, Hebei Province, and used for constructing the Δ*mcpC* strain. The *mcpC* gene deletion strain in this study was constructed using the *λ*-Red recombinase-mediated gene replacement method (Datsenko and Wanner, 2000). The bacteria were cultured in Luria-Bertani (LB) broth (Haibo Biotechnology Co., Ltd.) at 37°C unless otherwise specified. Human epithelial Caco-2 BBE cells and mouse macrophage RAW264.7 cells used in this study were provided by BeNa Culture Collection (Shanghai, China). Both cell types were cultured in DMEM (Thermo Fisher Scientific Co., Ltd.) supplemented with 10% fetal bovine serum (Thermo Fisher Scientific Co., Ltd.). Antibiotics were added as necessary, such as 50 μg/mL streptomycin and 50 U/mL penicillin, or 50 μg/mL gentamicin, in an incubator with 5% CO₂.

**Table 1:** Bacterial strains and plasmids used in this study.

Strains	Relevant characteristics	Sources
C50336	*Salmonella enterica* serovar *Enteritidis*, wild-type	This study
Δ*mcpC*::*cat*	A first recombination strain	This study
Δ*mcpC*	A second recombination strain	This study
Δ*mcpC + mcpC*	Δ*mcpC*-complemented strain	This study

Plasmids	Characteristics	Sources
pKD3	Template plasmid; FRT-*aphT*-FRT (containing chloramphenicol resistance gene)	Datsenko and Wanner (2000)
pKD46	Red recombinase expression plasmid blapBAD gam bet exopSC101 oriTS (containing ampicillin resistance gene)	Datsenko and Wanner (2000)
pCP20	FLP recombinase expression plasmid	Datsenko and Wanner (2000)

### Experimental animals and ethical statement

Kunming (KM) mice were obtained from Beijing Speifu Biotechnology Co., Ltd. All animal experiments were conducted in full compliance with international ethical standards and the Experimental Animal Regulation Ordinances (HPDST 2020-17) as stipulated by the Hebei Provincial Department of Science and Technology. The study protocol was reviewed and approved by the Animal Care and Use Committee of Hebei Normal University of Science and Technology.

### Construction of the *mcpC* deletion strain and complemented strain

C50336 (pKD46) was cultured in Luria-Bertani agar (LBA) containing 225 mg/mL L-arabinose at 30°C until the optical density (OD) at 600 nm reached 0.6–0.8. The cells were then washed three times with pre-chilled autoclaved ultrapure water to prepare electrocompetent cells. Using DH5α (pKD3) as a template, a homologous targeting fragment was amplified with specific primers P1 and P2 (Table 2). The purified fragment was electroporated into electrocompetent C50336 (pKD46) cells. Subsequently, 1 mL of LB broth was added, and the cells were incubated at 30°C for 2 h. The culture was then plated onto LB agar containing 50 μg/mL chloramphenicol (Cm). Colonies were identified using primers P3 and P4 (Table 2). Positive strains were cured of the pKD46 plasmid by incubation at 42°C and were designated as Δ*mcpC*::*cat*.

**Table 2:** Primers used for constructing the mutant and the complemented strain.

Primer	Sequence (5′–3′)
P1:	AGCAGCTCATGTTACTGGATGAAGAGGGGCGCTGGAGCCAGAGTTCGCAGAAAGAGCTGTGTGTAGGCTGGAGCTGCTTCG
P2:	CGCACGCGCCGCTTCAACCGCCGCGTTCAGCGCCAGAATATTGGTCTGAAAGGCAATGGCATATGAATATCCTCCTTAG
P3:	CGCTCTGTCTTTGTTTAGCCTTGA
P4:	ATCCCTTCCTGAGTCTGACTGGTT
P5:	GAAAATATGTTTTTGCATAACATTAAAA
P6:	TTAAGCGGGCTGCGTGTCCTCTTCGCGGA

To remove the Cm cassette, the pCP20 plasmid was introduced into Δ*mcpC*::*cat* via electroporation. Mutants were identified using primers P3 and P4, and positive strains were cured of the pCP20 plasmid by incubation at 42°C, resulting in the final Δ*mcpC* strain.

For the construction of the complemented strain, the open reading frame of the *mcpC* gene was amplified using primers P5 and P6 (Table 2). The purified PCR product was cloned into the pMD-19 T vector [Takara Biomedical Technology (Beijing) Co., Ltd.], resulting in the recombinant plasmid pMD-19 T-*mcpC*. This plasmid was then introduced into Δ*mcpC* by electroporation, and transformants were confirmed using primers P5 and P6. The complemented strain was designated as Δ*mcpC* + *mcpC*.

### *In vitro* stress simulation experiments

Overnight cultures of C50336, Δ*mcpC* and Δ*mcpC +* Δ*mcpC* were washed three times with physiological saline and enumerated using the traditional plate counting method to determine the initial bacterial count. The bacterial suspensions were then subjected to static incubation under various stress conditions: physiological saline at pH 3.5, physiological saline at pH 10, and at 42°C for 1 h. Additionally, the bacterial suspensions were exposed to physiological saline containing 10 mmol/L H_2_O_2_ for 10 min. After stress treatment, bacterial counts were determined, representing the post-stress bacterial count. The survival rate of each strain under different conditions was calculated as follows: survival rate = (post-stress bacterial count)/(initial bacterial count).

### Biofilm formation assay

The crystal violet (CV) staining method was used to assess biofilm formation (Zhang et al., 2020). Bacterial suspensions of C50336, Δ*mcpC*, and Δ*mcpC* + *mcpC* were inoculated at a 1:100 ratio into glass tubes containing 6 mL of LB broth and incubated statically at 28°C for 3 days. The tubes were washed 2–3 times with PBS, fixed with anhydrous methanol for 15 min, and stained with 2% CV for 15 min. The presence and thickness of stained bacterial rings on the tube walls were observed.

For quantitative analysis, a 96-well plate assay was performed. Each well was inoculated with 150 μL of bacterial suspension and stained as described above. After staining, 200 μL of anhydrous ethanol was added to each well to dissolve the CV. Absorbance at 570 nm was measured, and the experiment was repeated three times.

To detect two major components of the biofilm, curli fimbriae and cellulose, 5 μL of bacterial suspension from C50336, Δ*mcpC*, and Δ*mcpC* + *mcpC* was spotted onto salt-free LB agar containing 160 mg/L Congo red and 10 mg/L Coomassie brilliant blue (for curli detection) or onto agar containing 200 mg/L Calcofluor White Stain (CWS) for cellulose detection. Plates were incubated at 28°C for 2 days. Colony color, morphology, and fluorescence intensity under UV light (366 nm) were observed (Dong et al., 2011).

### Motility assay

Bacterial motility was assessed using semi-solid agar plates (0.3% agar). Briefly, 5 μL of overnight bacterial culture was carefully inoculated into the center of the semi-solid agar plate by gently stabbing it with a pipette tip to avoid agitation. Plates were incubated upright at 37°C for 5–6 h. Motility was determined by observing the migration of bacteria from the inoculation site toward the periphery of the plate. The experiment was repeated three times (Eakley et al., 2011).

### Adhesion, invasion, and intracellular survival assays

Bacterial suspensions of C50336, Δ*mcpC*, and Δ*mcpC* + *mcpC* were washed three times with PBS and enumerated using CFU calculations from serial dilutions on agar plates. Caco-2 BBE cells were seeded into 12-well plates at a density of 10^5^ CFU/well and cultured overnight in antibiotic-free DMEM supplemented with 10% FBS to achieve 80% confluence. The cells were washed three times with sterile PBS, and 1 mL of bacterial suspension was added to each well at a multiplicity of infection (MOI) of 100. Plates were centrifuged at 1,000 rpm for 5 min and incubated in a 37°C, 5% CO₂ incubator for 1 h (Xiong et al., 2023).

#### Adhesion assay

After a 1-h incubation, the cells were washed three times with PBS and lysed with 1% Triton X-100 for 8 min. The cell lysates were serially diluted and plated on SS agar for bacterial enumeration. Adhesion rate = (number of adhered bacteria/number of bacteria in the inoculum per well) × 100%.

#### Invasion assay

After a 1-h incubation, the cells were washed three times with PBS and further incubated for 1 h in DMEM containing gentamicin (100 μg/mL) to kill extracellular bacteria. The cells were then lysed with 1% Triton X-100 for 8 min, and the lysates were plated for bacterial enumeration. Invasion rate = (number of intracellular bacteria/number of bacteria in the inoculum per well) × 100%.

For the intracellular survival assay, RAW264.7 cells were seeded at a density of 10^5^ cells per well into two 12-well plates. Cells were infected with bacteria at an MOI of 100 and incubated at 37°C with 5% CO₂ for 2 h. Non-adherent and non-invaded bacteria were removed by washing the cells twice with PBS. The cells were then incubated for 1 h in DMEM containing 100 μg/mL gentamicin to eliminate remaining extracellular bacteria. The cells were lysed with 1% Triton X-100, and intracellular bacteria were enumerated, representing the bacterial count at 3 h post-infection. For the second plate, the cells were further incubated in DMEM containing 10 μg/mL gentamicin for 20 h. Cells were then lysed with 1% Triton X-100, and intracellular bacteria were enumerated to represent the bacterial count at 23 h post-infection. Intracellular survival rate = (intracellular bacteria at 23 h/intracellular bacteria at 3 h) × 100%.

### Determination of LD_50_ in mice

Eighty KM mice aged 4 to 6 weeks were randomly divided into 16 groups (*n* = 5). The first five groups were intraperitoneally (i.p.) injected with C50336 at doses of 2 × 10^7^, 2 × 10^6^, 2 × 10^5^, 2 × 10^4^, and 2 × 10^3^ CFU/mouse, respectively. The second five groups were i.p. injected with Δ*mcpC* at doses of 3.8 × 10^9^, 3.8 × 10^8^, 3.8 × 10^7^, 3.8 × 10^6^, and 3.8 × 10^5^ CFU/mouse, respectively. The third five groups were i.p. injected with Δ*mcpC + mcpC* at doses of 2 × 10^7^, 2 × 10^6^, 2 × 10^5^, 2 × 10^4^, and 2 × 10^3^ CFU/mouse, respectively. The remaining group was i.p. injected with an equal volume of PBS. The mortality of the mice was observed and recorded over a 14-day period following the injection (Yin et al., 2019).

The LD_50_ value was calculated using the formula of log_10_ (50% endpoint) = A + (B × C), where A = log_10_ (infectious dose showing a mortality next below 50%), B = difference of logarithms = [50% − (mortality at infectious dose next below 50%)]/[(mortality next above 50%) − (mortality next below 50%)], and C = log_10_ (difference between serial infectious doses used in challenge studies) (Park et al., 2022).

### RNA extraction and quantitative real-time PCR

To further investigate the effect of the *mcpC* gene on the virulence of *SE*, quantitative real-time PCR (qPCR) was employed to analyze the expression levels of *SE* virulence-related genes after the deletion of the *mcpC* gene. The procedure was as follows: RNA was extracted using a bacterial RNA extraction kit (Beijing Aidlab Biotechnologies Co., Ltd.), and genomic DNA was removed via DNase I treatment. The RNA was then reverse-transcribed into cDNA using a reverse transcription kit (Bohang Biotechnology Co., Ltd.). This cDNA was used as the template for qPCR analysis with the SYBR Green dye method. The UltraSYBR Mixture used in this method is from Jiangsu Cowin Biotech Co, Ltd. Virulence factor-related genes and their primers were selected based on previously reported literature (Table 3), with the *16S rRNA* gene serving as the internal control (Frye et al., 2006; Upadhyaya et al., 2013). The qPCR thermal cycling conditions were as follows: initial denaturation at 95°C for 10 min, followed by 40 cycles of 95°C for 15 s and 60°C for 1 min.

**Table 3:** Primers used for qPCR.

Gene	Sequence (5′–3′)
*fimD*-F	CGCGGCGAAAGTTATTTCAA
*fimD*-R	CCACGGACGCGGTATCC
*flgG*-F	GCGCCGGACGATTGC
*flgG*-R	CCGGGCTGGAAAGCATT
*prot6E*-F	GAACGTTTGGCTGCCTATGG
*prot6E*-R	CGCAGTGACTGGCATCAAGA
*csgA*-F	AATGCCACCATCGACCAGTG
*csgA*-R	CAAAACCAACCTGACGCACC
*csgD*-F	GCCTCATATTAACGGCGTG
*csgD*-R	AGCGGTAATTTCCTGAGTGC
*bcsA*-F	GCCCAGCTTCAGAATATCCA
*bcsA*-R	TGGAAGGGCAGAAAGTGAAT
*ompR*-F	TGTGCCGGATCTTCTTCCA
*ompR*-R	CTCCATCGACGTCCAGATCTC
*hflK*-F	AGCGCGGCGTTGTGA
*hflK*-R	TCAGACCTGGCTCTACCAGATG
*tatA*-F	AGTATTTGGCAGTTGTTGATTGTTG
*tatA*-R	ACCGATGGAACCGAGTTTTTT
*lrp*-F	TTAATGCCGCCGTGCAA
*lrp*-R	GCCGGAAACCAAATGACACT
*sipA*-F	CAGGGAACGGTGTGGAGGTA
*sipA*-R	AGACGTTTTTGGGTGTGATACGT
*sipB*-F	GCCACTGCTGAATCTGATCCA
*sipB*-R	CGAGGCGCTTGCTGATTT
*pipB-*F	GCTCCTGTTAATGATTTCGCTAAAG
*pipB-*R	GCTCAGACTTAACTGACACCAAACTAA
*invH-*F	CCCTTCCTCCGTGAGCAAA
*invH-*R	TGGCCAGTTGCTCTTTCTGA
*mgtC*-F	CGAACCTCGCTTTCATCTTCTT
*mgtC*-R	CCGCCGAGGGAGAAAAAC
*sodC*-F	CACATGGATCATGAGCGCTTT
*sodC*-R	CTGCGCCGCGTCTGA
*orf245*-F	CAGGGTAATATCGATGTGGACTACA
*orf245*-R	GCGGTATGTGGAAAACGAGTTT
*rfbH-*F	ACGGTCGGTATTTGTCAACTCA
*rfbH-*R	TCGCCAACCGTATTTTGCTAA
*xthA*-F	CGCCCGTCCCCATCA
*xthA*-R	CACATCGGGCTGGTGTTTT
*mrr1*-F	CCATCGCTTCCAGCAACTG
*mrr1*-R	TCTCTACCATGAACCCGTACAAATT
*16S rRNA*-F	CCAGGGCTACACACGTGCTA
*16S rRNA*-R	TCTCGCGAGGTCGCTTCT

### Bacterial colonization and persistence in organs

Thirty-five 4–6-week-old KM mice were randomly divided into two groups: the C50336 infection group (Group A, *n* = 20) and the Δ*mcpC* infection group (Group B, *n* = 15). Mice in Group A were i.p. injected with the wild-type strain C50336, while mice in Group B were i.p. injected with the gene-deleted strain Δ*mcpC*. The injection dose for both groups was 2 × 10^5^ CFU/mouse. On days 3, 7, and 14 post-infection, five mice were randomly selected from each group and euthanized. Under sterile conditions, the liver, spleen, and cecum were collected, weighed, and homogenized in 1 mL of sterile PBS. Serial dilutions of the homogenate were plated on SS agar for bacterial enumeration. The bacterial load in each organ was calculated using the formula log_10_ CFU/g (Negi et al., 2007; Milanez et al., 2018).

### Immunization with Δ*mcpC* in mice

Twenty-four 6 to 8-week-old female KM mice were randomly divided into two groups: the immunized group (*n* = 12) and the control group (*n* = 12). The immunized group received an oral dose (p.o.) of 2 × 10^7^ CFU/mouse of the Δ*mcpC* strain. The procedure was as follows: after fasting and water deprivation for 6 h, each mouse was orally administered 100 μL of 5% NaHCO₃ to neutralize gastric acid. Two hours later, oral inoculation was performed using a gavage needle. On day 14 post-immunization (dpi), a booster immunization with the same dose was administered. The control group received an equivalent volume of PBS via oral administration.

### Detection of IgG and IgA

The indirect ELISA method was employed to detect *SE*-specific IgG and IgA in the serum and feces of immunized mice. The strain C50336 was cultured to the logarithmic growth phase, washed twice with PBS, and resuspended. The suspension was ultrasonically lysed for 30 min at 4°C, followed by centrifugation at 8,000 rpm for 10 min. The supernatant was collected and stored at −80°C for use as the coating antigen (Ji et al., 2022). At 14 dpi and 28 dpi, blood samples were randomly collected from three mice in the immunized group and three in the control group. The blood was centrifuged to separate the serum, which was stored at −80°C for IgG detection. Fecal samples were also collected from the same three mice. Five fecal pellets from each mouse were suspended in 0.5 mL of extraction buffer containing 0.1 mg/mL soybean trypsin inhibitor (Shanghai Yuanye Bio-Technology Co., Ltd.), 10 mg/mL bovine serum albumin (Biofroxx), and 30 mM EDTA disodium (pH 7.6). After homogenization and centrifugation, the supernatant was collected and stored at −80°C for IgA detection (Nandre et al., 2011; Pasquevich et al., 2011).

Using a checkerboard titration method, the optimal antigen coating concentration and sample dilution were determined. The coating antigen (500 ng/100 μL) was added to a 96-well microtiter plate and incubated overnight at 4°C. Blocking was performed using 5% skim milk (prepared in PBS containing 0.05% Tween 20, PBST) at 37°C for 4 h (200 μL/well). After removing the blocking solution, the plate was washed three times with PBST. Serum samples diluted 1:400 or undiluted fecal supernatant (100 μL/well) were added and incubated at 37°C for 1 h. The plate was then washed three times with PBST. HRP-conjugated goat anti-mouse IgG (diluted 1:10,000, Beijing Solarbio Science & Technology Co., Ltd.) or HRP-conjugated goat anti-mouse IgA (diluted 1:10,000, Beijing Pulilai Gene Technology Co., Ltd.) was added at 100 μL/well and incubated at 37°C for 1 h. Following another washing step, 100 μL of TMB substrate solution (Beijing Solarbio Science & Technology Co., Ltd.) was added to each well and incubated at 37°C for 10 min. The reaction was terminated by adding 50 μL of 2 M H_2_SO_4_ to each well, and the OD_450nm_ was measured (Park et al., 2022).

### Lymphocyte proliferation assay

The C50336 bacterial antigen was prepared following the previously described method and used as a stimulant for the lymphocyte proliferation assay. At 14 dpi and 28 dpi, three mice from each group were euthanized. The spleens of the immunized mice were aseptically isolated, homogenized, and filtered through a 70 μm cell strainer (Beijing Labgic Technology Co., Ltd.) to obtain spleen cells. Red blood cells were lysed using a red blood cell lysis buffer (Beijing Solarbio Science & Technology Co., Ltd.). The spleen lymphocytes were suspended in RPMI Medium 1,640 (Thermo Fisher Scientific Co., Ltd.) supplemented with 10% fetal bovine serum (FBS), 50 U/mL penicillin, and 50 μg/mL streptomycin. Cell viability was assessed using the trypan blue exclusion test, and cells were counted using a hemocytometer.

In a 96-well tissue culture plate, 10^5^ cells/100 μL of cell suspension were added to each well. For the stimulation group, bacterial antigen was added at a final concentration of 7.5 μg/mL (11 μL/well). For the non-stimulation group, 11 μL of RPMI Medium 1,640 containing 10% FBS, 50 U/mL penicillin, and 50 μg/mL streptomycin was added to each well. A medium-only control group was also included (111 μL/well). The 96-well tissue culture plates were incubated at 37°C in a humidified environment with 5% CO₂ for 72 h. Lymphocyte proliferation was measured using the MTT Cell Proliferation and Cytotoxicity Assay Kit (Shanghai Beyotime Biotechnology Co., Ltd.). Absorbance was measured at 450 nm, and the stimulation index (SI) was calculated as follows (Lin et al., 2017):


$$\begin{array}{l} SI=\left(\begin{array}{l} \mathrm{Mean}\;OD\;\mathrm{value of stimulation group}-\\ \mathrm{Mean}\;OD\;\mathrm{value of medium}-\mathrm{only group} \end{array}\right)/\\ \left(\begin{array}{l} \mathrm{Mean}\;OD\;\mathrm{value of non}-\\ \mathrm{stimulation group}-\\ \mathrm{Mean}\;OD\;\mathrm{value of}\;\\ \mathrm{medium}-\mathrm{only group} \end{array}\right) \end{array}$$

### The expression of cytokines in the spleen

qPCR was used to evaluate the expression levels of cytokines *IL-1β*, *IL-2*, *IL-4*, *IL-6*, *IL-10*, *IFN-γ*, and *TNF-α* in the spleen of immunized mice. At 14 dpi and 28 dpi, three mice from each group were randomly selected. Spleens were aseptically isolated, and total RNA was extracted using the TriQuick Total RNA Extraction Kit (Beijing Solarbio Science & Technology Co., Ltd.). DNA was removed by DNase I treatment, and RNA was reverse transcribed into cDNA using a reverse transcription kit (Bohang Biotechnology Co., Ltd.). Samples were stored at −80°C until use. RT-PCR for gene expression studies was performed using UltraSYBR Mixture (Jiangsu Cowin Biotech Co, Ltd., China).

The primers used for the qPCR are shown in Table 4. Cytokine expression levels were normalized to the internal control gene *gapdh* and *β-actin* and calculated using the $2^{-\Delta \Delta C_{T}}$ method. Thermal cycling conditions: initial denaturation at 95°C for 10 min, followed by 40 cycles of 95°C for 15 s and 60°C for 1 min (Wang et al., 2021; Kang et al., 2022).

**Table 4:** Primers used for the qPCR amplification of cytokines.

Primers	Sequence (5′–3′)
*IL-1β*-F	GACTGTTTCTAATGCCTTCCC
*IL-1β*-R	ATGGTTTCTTGTGACCCTGA
*IL-2*-F	TGAGCAGGATGGAGAATTACAGG
*IL-2*-R	GTCCAAGTTCATCTTCTAGGCAC
*IL-4*-F	GGTCTCAACCCCCAGCTAGT
*IL-4*-R	GCCGATGATCTCTCTCAAGTGAT
*IL-6*-F	TAGTCCTTCCTACCCCAATTTCC
*IL-6*-R	TTGGTCCTTAGCCACTCCTTC
*IL-10*-F	CTTACTGACTGGCATGAGGATCA
*IL-10*-R	GCAGCTCTAGGAGCATGTGG
*IFN-γ*-F	ATGAACGCTACACACTGCATC
*IFN-γ*-R	CCATCCTTTTGCCAGTTCCTC
*TNF-α*-F	CCCTCACACTCAGATCATCTTCT
*TNF-α*-R	GCTACGACGTGGGCTACAG
*GAPDH*-F	AGGTCGGTGTGAACGGATTTG
*GAPDH*-R	TGTAGACCATGTAGTTGAGGTCA
*β-Actin-F*	TTCAACACCCCAGCCATG
*β-Actin-F*	CCTCGTAGATGGGCACAGT

### Immune protection assessment in mice

Forty 6 to 8-week-old KM female mice were randomly divided into four groups (n = 10): the immunization groups (Group A and Group B), the challenge group (Group C), and the control group (Group D). Mice in Group A were orally administered 2 × 10^7^ CFU/mouse of Δ*mcpC*, with a booster immunization at the same dose at 14 dpi. Mice in Group B were orally administered 2 × 10^6^ CFU/mouse of Δ*mcpC*, also with a booster immunization at the same dose at 14 dpi. Mice in Groups C and D were orally administered an equivalent volume of PBS. At 28 dpi, mice in the immunization groups (Groups A and B) and the challenge group (Group C) were i.p. injected with 2 × 10^7^ CFU/mouse of C50336, while mice in the control group (Group D) were i.p. injected with an equivalent volume of PBS. For 14 days post-challenge, mouse mortality was recorded daily, and the relative survival rate was calculated as follows:


$$\begin{array}{l} \mathrm{Relative survival rate}=\left(\begin{array}{l} \mathrm{Mortality rate of the}\;\\ \mathrm{control group}-\\ \mathrm{Mortality rate of the}\;\\ \mathrm{experimental group} \end{array}\right)/\\ \mathrm{Mortality rate of the}\;\\ \mathrm{control group}\times 100\% \end{array}$$

### Statistical analysis

Data are expressed as the mean ± standard error of the mean (SEM). All statistical analysis were two-way ANOVA and post-test. Differences between two samples were evaluated using Student’s *t*-test. Significant differences are indicated with an asterisk (*), where ^*^p < 0.05, ^**^p < 0.01, and ^***^p < 0.001 are considered to represent statistically significant differences in mean values. Statistical analyses were conducted using IBM SPSS Statistics 26.

## Results

### The Δ*mcpC* mutation results in a reduced stress defense capacity

**Figure 1:**
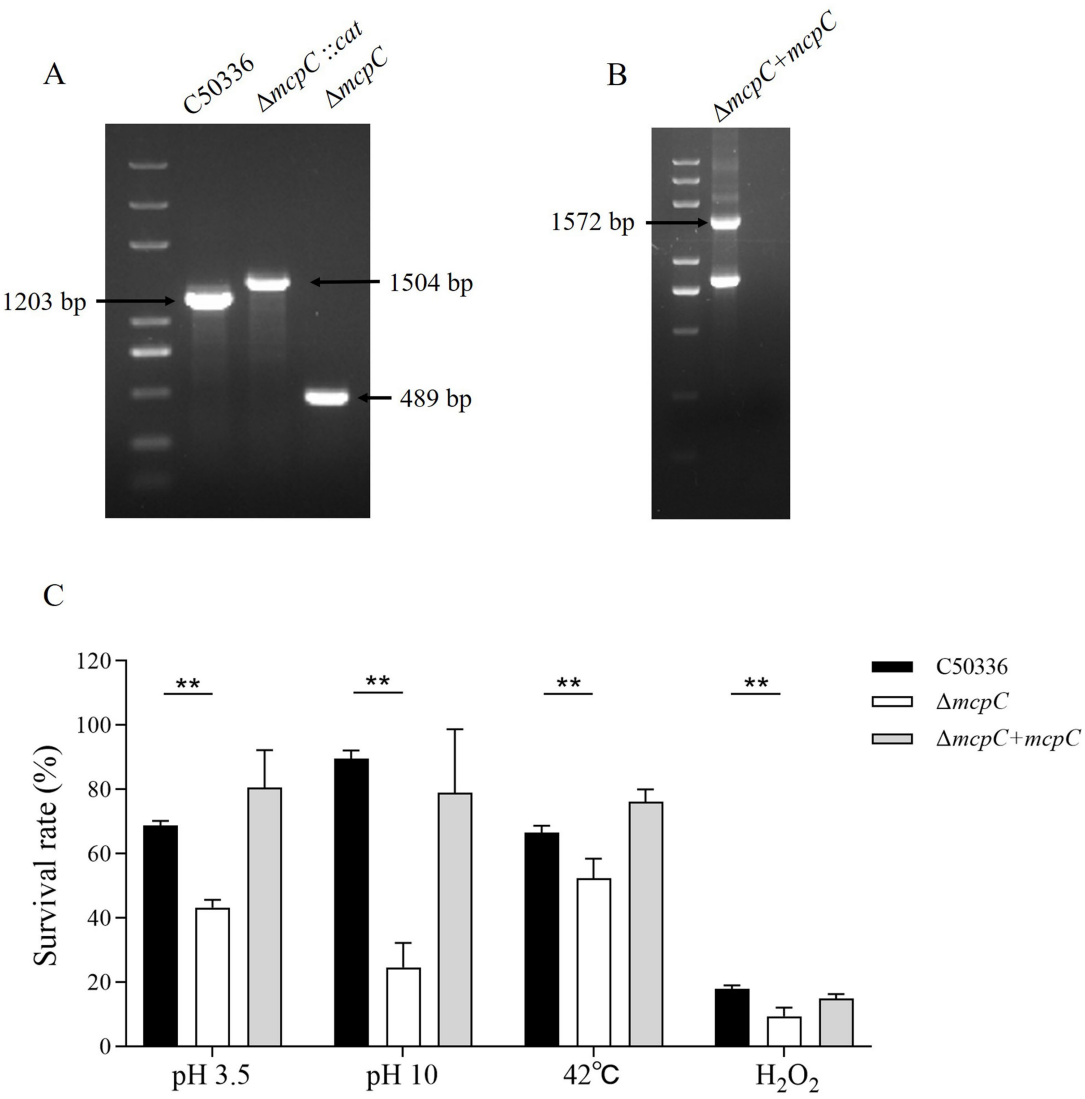
**(A)** PCR verification of the *mcpC* gene deletion strain. The PCR product of C50336 has a length of 1,203 bp, the product of Δ*mcpC*::*cat* has a length of 1,504 bp, the product of Δ*mcpC* has a length of 489 bp. **(B)** PCR verification of Δ*mcpC* + *mcpC*. The PCR product has a length of 1,572 bp. **(C)** The survival rate of C50336, Δ*mcpC* and Δ*mcpC* + *mcpC* under various environmental stresses. The data represents the average of 3 replicates.

Genomic DNA from the strains C50336, Δ*mcpC*::*cat*, and Δ*mcpC* was used as templates for PCR identification with primers P3 and P4. The results (Figure 1A) showed bands at 1203 bp, 1,506 bp, and 489 bp, respectively, consistent with the expected sizes, indicating the successful knockout of the *mcpC* gene. Genomic DNA from the suspected complemented strain was used as a template, and primers P5 and P6 were employed for PCR identification. The results (Figure 1B) revealed a target band at approximately 1,572 bp, matching the expected size, confirming the successful construction of the *mcpC* complemented strain, designated as Δ*mcpC* + *mcpC*.

To investigate whether the *mcpC* gene affects the resistance of *SE* to various environmental stresses, the survival rates of C50336 and Δ*mcpC* were compared under conditions of pH 3.5, pH 10, 42°C, and 10 mmol/L H₂O₂. The results showed that (Figure 1C), compared to C50336, the survival rate of Δ*mcpC* was significantly reduced under all tested conditions, indicating that the deletion of the *mcpC* gene weakens *SE*’s resistance to acid, alkaline, thermal, and oxidative stresses.

### The *mcpC* gene does not affect biofilm formationand drug resistance

**Figure 2:**
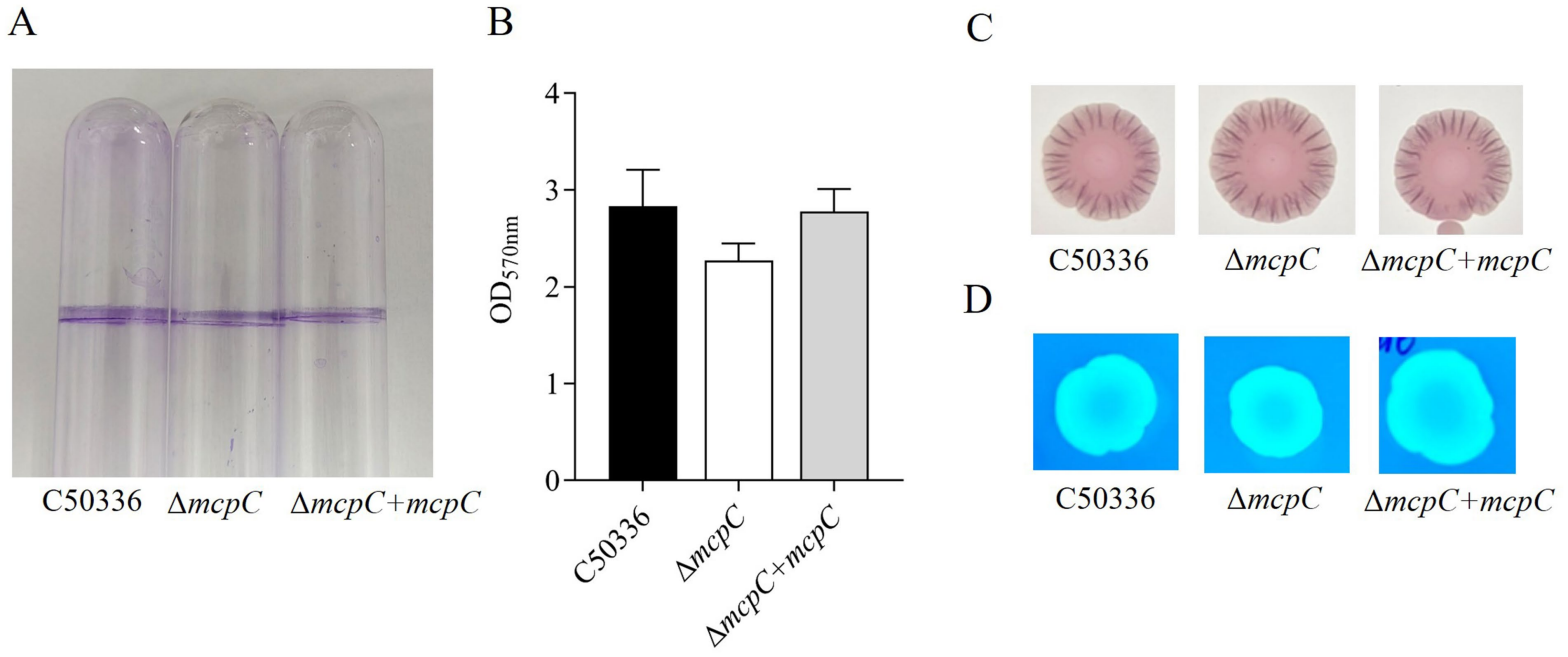
**(A)** Detection of biofilm formation in glass test tubes. **(B)** Qualitative detection of biofilm formation in microtiter plates, with absorbance measured at 570 nm. **(C)** Curli formation detection. **(D)** Cellulose formation detection.

The biofilm formation ability of strains C50336, Δ*mcpC*, and Δ*mcpC* + *mcpC* was evaluated. Tube assay results showed no significant differences in biofilm formation ability among the three strains (Figure 2A). Quantitative analysis using a 96-well plate assay also indicated no significant differences in biofilm formation among the strains (Figure 2B).

Detection of curli fimbriae, a major component of biofilms, revealed that all three strains formed red, rough colonies (Figure 2C), indicating their ability to produce curli fimbriae. Cellulose production analysis showed that colonies of all three strains exhibited the same fluorescence intensity under UV light (Figure 2D), confirming their ability to produce cellulose. These findings demonstrate that the *mcpC* gene does not affect the biofilm formation ability of *SE*.

### The Δ*mcpC* mutation results in motility reduction

**Figure 3:**
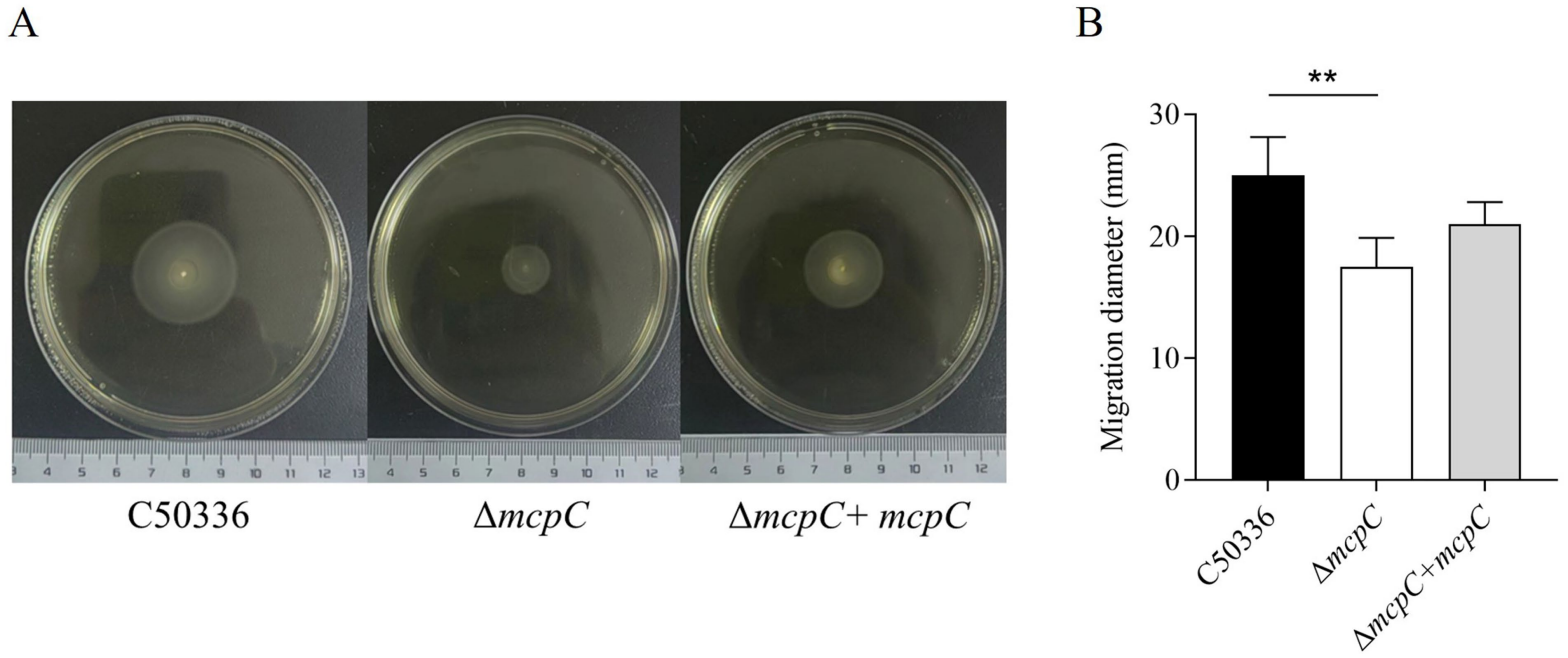
**(A)** The images shown are representatives of several independent assays. **(B)** The motility of the strains was evaluated on 0.3% agar plates, measured after 5 h of incubation. The data represents the average of 3 replicates.

To assess whether the deletion of the *mcpC* gene affects the motility of *SE*, motility assays were conducted. The results showed that the average migration diameter of the Δ*mcpC* strain (17.5 mm) was significantly smaller than that of the wild-type C50336 strain (25 mm) and the complemented strain Δ*mcpC* + *mcpC* (21 mm) (Figure 3). These findings indicate that the deletion of the *mcpC* gene impairs the motility of *SE*.

### Removal of *mcpC* weakens the invasion and intracellular survival of *SE*

**Figure 4:**
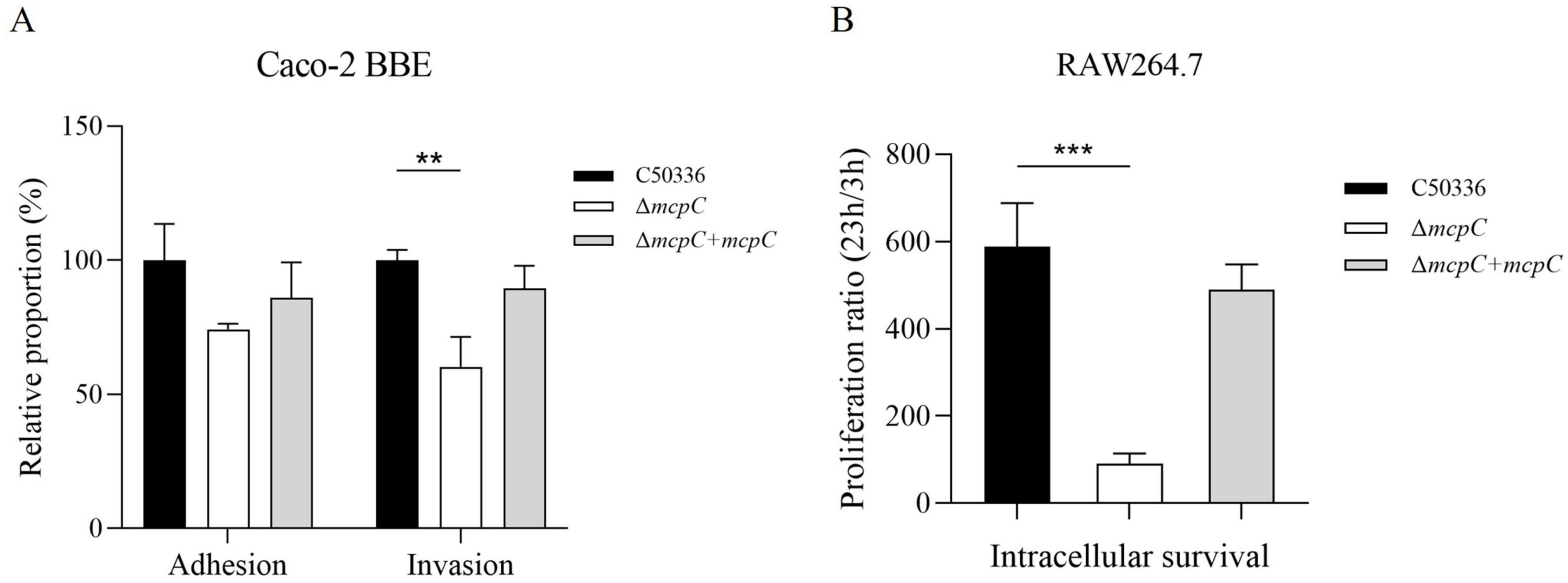
**(A)** Adhesion and invasion of bacteria in Caco-2 cells. **(B)** Intracellular survival in RAW264.7 cells. The results of the adhesion and invasion assays are presented as a ratio to the C50336 (the results of C50336 were considered as 100%). The data represents the average of 3 replicates.

The adhesion and invasion abilities of the Δ*mcpC* strain toward intestinal epithelial cells were assessed using human colorectal adenocarcinoma cells Caco-2. As shown in Figure 4A, the adhesion rate of Δ*mcpC* was comparable to that of the wild-type C50336 strain, but its invasion rate was significantly reduced.

The intracellular survival of Δ*mcpC* was evaluated using mouse-derived macrophages RAW264.7. The results revealed a significantly lower survival rate of Δ*mcpC* within macrophages compared to C50336 (Figure 4B). These findings indicate that the *mcpC* gene is critical for the invasion and intracellular survival capabilities of *SE*.

### Δ*mcpC* exhibits increased LD_50_ in mice

The virulence of C50336 and Δ*mcpC* strains was evaluated in mice via i.p. injection. Mice infected with Δ*mcpC* began to die on day 2, whereas those infected with C50336 showed mortality starting on day 3. In contrast, the control group exhibited no mortality and maintained normal behavior, including smooth fur, good mental state, and an absence of symptoms such as shivering, hunching, eye crusting, or disheveled fur.

The calculated LD_50_ values (Table 5) were 6.3 × 10^5^ CFU/mouse for C50336 and 1.9 × 10^7^ CFU/mouse for Δ*mcpC*, with the LD_50_ of Δ*mcpC* being approximately 30 times higher than that of C50336 (1.9 × 10^7^/6.3 × 10^5^ ≈ 30). The LD_50_ of the gene-complemented strain Δ*mcpC + mcpC* is 1.6 × 10^6^ CFU/mouse. The results suggest that the deletion of the *mcpC* gene significantly attenuates the virulence of *SE*.

**Table 5:** LD_50_ of C50336, Δ*mcpC* and Δ*mcpC + mcpC* in KM mice.

Strain	Inoculation dose (CFU/mouse)	No. of deaths/total No. of mice	LD_50_
C50336	2 × 10^7^	5/5	6.3 × 10^5^
	2 × 10^6^	3/5	
	2 × 10^5^	2/5	
	2 × 10^4^	0/5	
	2 × 10^3^	0/5	
Δ*mcpC*	3.8 × 10^9^	5/5	1.9 × 10^7^
	3.8 × 10^8^	5/5	
	3.8 × 10^7^	3/5	
	3.8 × 10^6^	1/5	
	3.8 × 10^5^	0/5	
Δ*mcpC + mcpC*	2 × 10^7^	5/5	1.6 × 10^6^
	2 × 10^6^	2/5	
	2 × 10^5^	1/5	
	2 × 10^4^	0/5	
	2 × 10^3^	0/5	

### Removal of mcpC results in a down-regulation of the multiple virulence gene expressionof in *SE*

**Figure 5:**
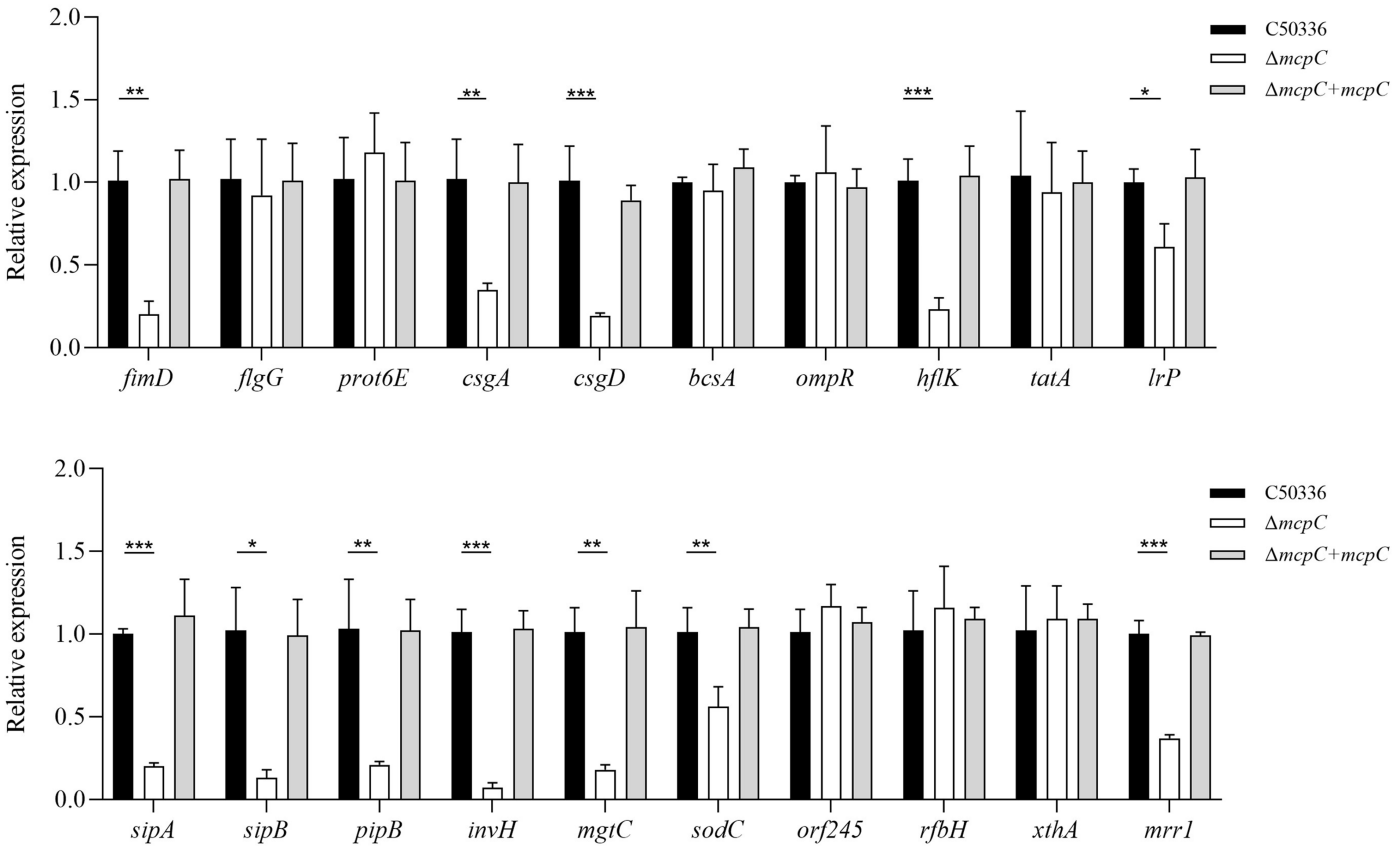
The expression levels of virulence genes in C50336, Δ*mcpC* and Δ*mcpC* + *mcpC* were detected by using qPCR, with *16S rRNA* as the housekeeping gene. The data represents the average of 3 replicates.

To investigate the mechanisms underlying the attenuated virulence of *SE* caused by the deletion of the *mcpC* gene, this study employed qPCR to examine the expression levels of various virulence genes. The results revealed that the deletion of *mcpC* significantly downregulated the expression of genes associated with bacterial motility (*fimD*), biofilm formation (*csgA*, *csgD*), cell membrane and cell wall integrity (*hflK*, *lrp*), the type III secretion system (T3SS) (*sipA*, *sipB*, *pipB*), adhesion and invasion (*invH*), intracellular survival (*mgtC*, *sodC*), and nucleic acid exonuclease/endonuclease activity (*mrr1*) (Figure 5). These findings suggest that *mcpC* may regulate the expression of multiple virulence genes, thereby contributing to the overall virulence of *SE*.

### Removal of *mcpC* results in reduced colonization and persistence of bacteria in the organ

Organ bacterial load is another critical indicator of *SE* virulence. In this study, the bacterial loads in the liver, spleen, and cecum of mice infected with C50336 and Δ*mcpC* were assessed. After homogenization of the organs, bacterial counts were measured.

**Figure 6:**
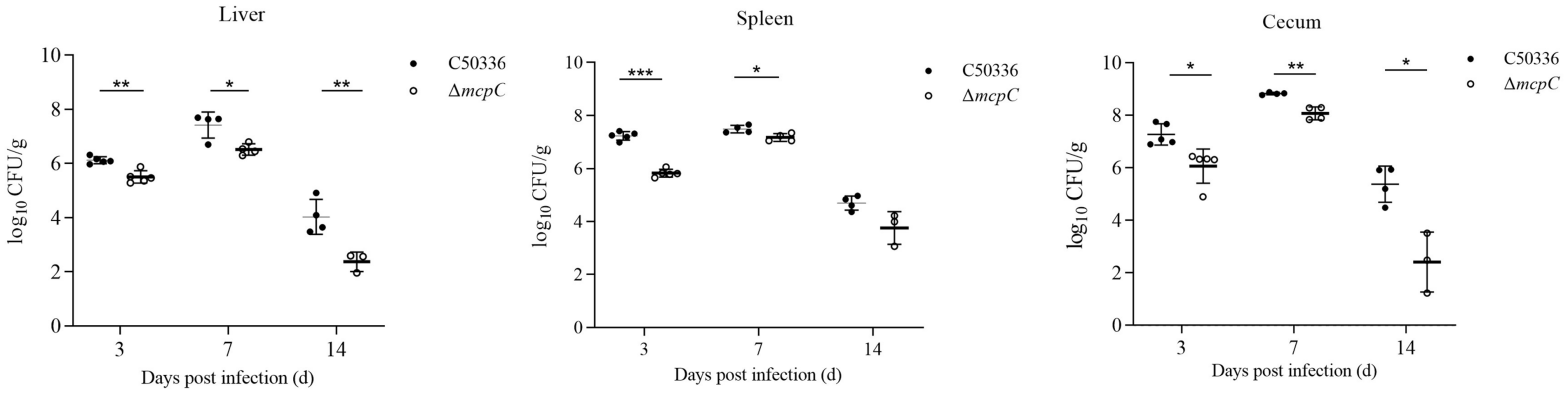
The colonization and persistence of C50336 and Δ*mcpC* colonization in liver, spleen and cecum after challenge. Values are represented as log_10_ CFU/g sample. Negative samples are shown as 0 CFU/g; all samples from the control group were negative.

As shown in Figure 6, bacteria were isolated from the liver, spleen, and cecum on days 3, 7, and 14 post-infection. The colonization levels of both C50336 and Δ*mcpC* peaked on day 7 and subsequently declined. Notably, compared to C50336, Δ*mcpC* exhibited significantly lower bacterial loads in the liver, spleen, and cecum on day 3 and day 7; in the liver and cecum on day 14.

These findings indicate that while Δ*mcpC* can still colonize organs, its colonization ability in certain organs is significantly reduced, suggesting that the deletion of the *mcpC* gene diminishes the organ bacterial load and virulence of *SE*.

### Δ*mcpC* can induce mucosal, humoral, and cellular immune responses

**Figure 7:**
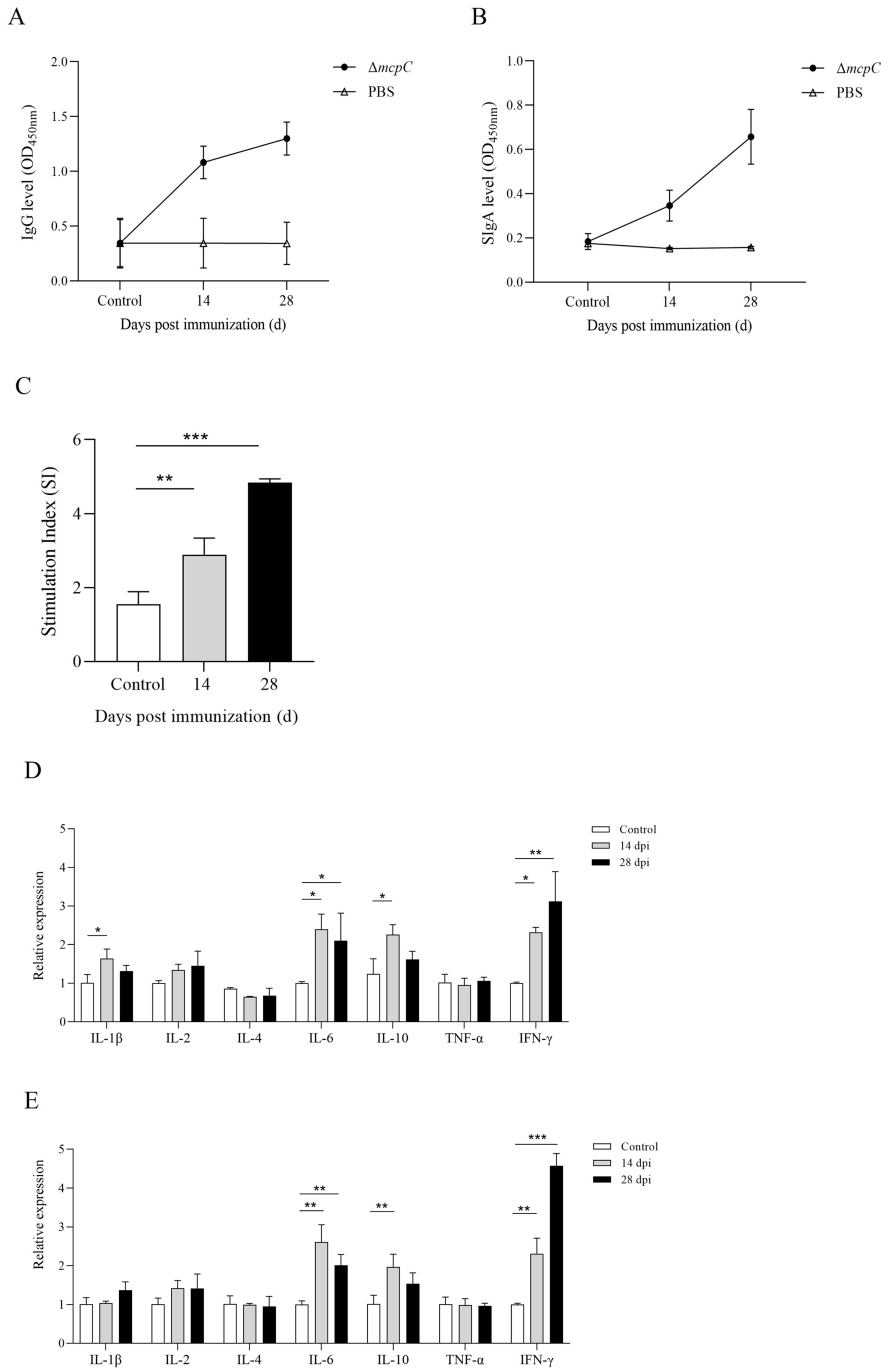
Production of *Salmonella*-specific antibodies in mouse sera and fecal. Levels of **(A)** IgG, **(B)** SIgA. **(C)** The lymphocyte proliferation was assessed at 14 dpi and 28 dpi using soluble antigen. At each time point, the mean stimulation index (SI) of the vaccinated group was significantly higher than that of the control group. **(D,E)** The expression level of cytokines *IL-1β*, *IL-2*, *IL-4*, *IL-6*, *IL-10*, *TNF-α* and *IFN-γ*. The internal control gene were *gapdh* and *β-actin*, respectively.

To evaluate the ability of Δ*mcpC* to induce specific humoral and mucosal immune responses, serum IgG and fecal SIgA levels were measured in immunized mice using indirect ELISA. The results showed that at 14 dpi and 28 dpi, mice immunized with Δ*mcpC* exhibited significantly higher serum IgG levels compared to the control group (Figure 7A). Additionally, elevated SIgA levels were detected in fecal samples (Figure 7B). Both serum IgG and fecal SIgA levels increased significantly following the second immunization compared to the first. These findings suggest that Δ*mcpC* effectively induces robust specific humoral and mucosal immune responses, which are enhanced with repeated immunizations.

Lymphocyte proliferation assays are a primary method for evaluating cellular immune responses. In this study, a lymphocyte proliferation assay was conducted using C50336 antigens as stimulants. The results revealed that lymphocyte proliferation, measured by the stimulation index (SI), was significantly higher in mice immunized with Δ*mcpC* compared to the control group (Figure 7C). Moreover, after the booster immunization, lymphocyte proliferation levels further increased. These findings indicate that Δ*mcpC* induces a strong specific immune response, which is enhanced with repeated immunizations.

The expression of different cytokines reflects the strength and polarization of the immune response. At 14 dpi and 28 dpi, the expression levels of *IL-1β*, *IL-2*, *IL-4*, *IL-6*, *IL-10*, *TNF-α*, and *IFN-γ* were analyzed using qPCR. The results showed that (Figures 7D,E), compared to the control group, the expression levels of *IL-6*, *IL-10*, and *IFN-γ* significantly increased after the first immunization. Following the second immunization, *IL-6* and *IFN-γ* expression levels further increased, although *IL-6* remained similar to the levels observed after the first immunization, while *IFN-γ* continued to rise. These findings suggest that Δ*mcpC* effectively induces a strong immune response in mice.

### Δ*mcpC* immunization provides powerful protective immune protection for mice

**Figure 8:**
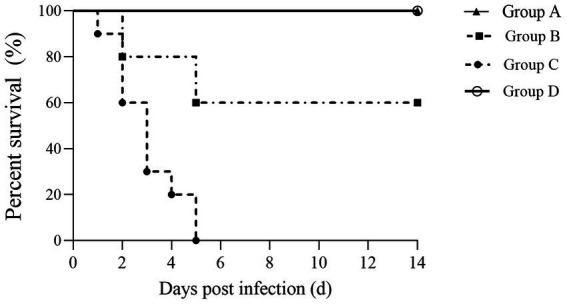
Survival curve. Female KM mice aged 6–8 weeks were p.o. inoculated with the Δ*mcpC* and i.p. injected with a lethal dose of C50336 at 28 dpi. The percent survival of mice was monitored daily.

To further evaluate the protective immunity conferred by Δ*mcpC*, this study challenged immunized mice with a virulent strain via i.p. injection, recorded survival rates, and plotted survival curves. The results showed that mice in Group A (oral immunization with a dose of 2 × 10^7^ CFU/mouse) exhibited 100% survival with no deaths recorded. In Group B (oral immunization with a dose of 2 × 10^6^ CFU/mouse), partial mortality was observed. In contrast, all mice in Group C (non-immunized, challenged group) succumbed within 5 days, while mice in Group D (non-immunized, non-challenged control group) showed no mortality (Figure 8).

The percentage survival of mice at 14 days post-challenge is summarized in Table 6. The results showed that Groups A and D achieved a relative survival rate of 100%, Group B exhibited a survival rate of 60%, while Group C had no survivors. These findings indicate that oral immunization with Δ*mcpC* offers robust protection against *SE* infection in mice.

**Table 6:** Protective effects of Δ*mcpC* in mice.

Group	Vaccination			Number	Challenge			Survivors/total	Percent survival (%)
	Strain	Route	Dose (CFU)		Strain	Route	Dose (CFU)		
A	Δ*mcpC*	p.o.	2 × 10^7^	10	C50336	i.p.	2 × 10^7^	10/10	100
B	Δ*mcpC*	p.o.	2 × 10^6^	10	C50336	i.p.	2 × 10^7^	6/10	60
C	PBS	p.o.	—	10	C50336	i.p.	2 × 10^7^	0/10	0
D	PBS	p.o.	—	10	PBS	i.p.	—	10/10	100

## Discussion

*SE* can cause subclinical infections in adult livestock and poultry, leading to bacterial shedding through feces into the external environment. This creates challenges in pathogen eradication, resulting in severe systemic infections and high mortality rates in animals. The use of antibiotics and vaccination are key strategies for controlling *Salmonella* infections. However, widespread antibiotic use has led to the emergence of multidrug-resistant strains and severe drug residue issues, posing a significant threat to public health. Therefore, there is an urgent need for an effective vaccine to control this important zoonotic pathogen. As an intracellular pathogen, the host’s cellular immune response plays a crucial role in limiting *SE* infections. Live attenuated vaccines are considered more effective than inactivated vaccines in combating both intestinal and systemic infections, as they can simultaneously stimulate robust cellular and humoral immune responses (Wang et al., 2022). In this study, a *mcpC* mutant strain of *SE* Δ*mcpC* exhibited significantly attenuated virulence and provided strong immune protection in mice.

During its pathogenesis, SE must overcome numerous adverse environmental conditions, including antimicrobial peptides, temperature and pH fluctuations, and nutrient limitations (Rana et al., 2021). The ability to survive under these diverse environmental conditions is a fundamental characteristic of SE virulence (Arunima et al., 2020). In this study, the deletion of the mcpC gene impairs SE’s ability to sense and respond to environmental changes, such as acid, alkali, heat, and oxidative stress. A large number of studies showed that Lon, CpxR, RfaL, and RpoS significantly affect the strain’s stress defense capacity, thereby influencing the bacteria’s pathogenicity (Kirthika et al., 2024; Badie et al., 2021). Thus, it is speculated that McpC helps *Salmonella* cope with challenges such as oxidative stress, high temperature, and hypoxia, and may influence the bacteria’s virulence.

Numerous studies have demonstrated that *Salmonella* can form biofilms on a variety of contact surfaces (Römling et al., 2003). These biofilms exhibit high resistance to antimicrobial agents and contribute to the increased virulence of *Salmonella*, facilitating the establishment of chronic infections (Merino et al., 2019). Biofilm formation allows *Salmonella* to persist in poultry farming environments and contaminate poultry meat and eggs, which remain major vehicles for foodborne *Salmonella* outbreaks. Curli fimbriae and cellulose are the primary components of *Salmonella* biofilms. The Δ*mcpC* was no significant reduction in the formation of biofilm, curli fimbriae, and cellulose. But the expression levels of biofilm-related genes, *csgA* and *csgD*, were significantly reduced. One possible explanation is that while the deletion of the *mcpC* gene downregulates certain biofilm-related genes, biofilm formation is regulated by the collective interaction of multiple genes.

Bacterial motility is closely associated with chemotaxis. The combination of motility and chemotaxis enables bacteria to detect and pursue nutrients, allowing them to reach and maintain their preferred colonization niches (Josenhans and Suerbaum, 2002). The level of motility plays a critical role in determining whether *Salmonella* can reach specific sites within the host, thereby influencing its virulence. We observed that the motility of the Δ*mcpC* was significantly reduced. The expression of the motility-associated gene *fimD was* down-regulated in Δ*mcpC*. These findings suggest that the deletion of the *mcpC* gene impairs the motility of *SE*, which in turn affects its pathogenic process. This hypothesis is supported by many studies, such as the finding that the c-di-GMP binding effector STM0435 regulates flagella synthesis, controls biofilm formation, and affects Salmonella virulence (Dai et al., 2024). In addition, the knockout of YeiE reduces the expression of flagella-related genes such as fliA, flgM, and fliD in *Salmonella enterica*, leading to a decrease in flagella formation, which affects the bacteria’s colonization of the intestines and its virulence (Westerman et al., 2021).

The pathogenic process of *Salmonella* involves adhesion and invasion of intestinal epithelial cells, survival and replication within host cells, and dissemination beyond the intestine. Invasion of host cells is a critical characteristic of *Salmonella* pathogenicity, while survival within macrophages is considered a more robust indicator of bacterial virulence (Fields et al., 1986; Eakley et al., 2011). We found that that the deletion of the *mcpC* gene impairs the invasion and intracellular survival capabilities of *SE*, both of which are closely linked to its virulence.

An ideal attenuated live vaccine strain should effectively withstand host-induced stress, provide robust protection against the target pathogen, and successfully colonize host lymphoid tissues, all while maintaining an avirulent profile (Pati et al., 2013). This study showed that the LD_50_ of Δ*mcpC* was significantly increased, and the expression of multiple virulence genes is down-regulated, indicating a reduction in its virulence. Following ingestion, *Salmonella* replicates in mucosa-associated lymphoid tissues, such as Peyer’s patches, and disseminates via mesenteric lymph nodes to systemic organs, including the spleen and liver. The cecum is also a primary colonization site for *SE* in poultry. Organ bacterial load is a critical indicator of *Salmonella* virulence. Our results showed that Δ*mcpC* exhibited significantly reduced bacterial loads in these organs compared to the wild-type C50336 strain, indicating that *mcpC* deletion diminishes the pathogenicity of *SE*. Collectively, these findings suggest that the Δ*mcpC* mutant strain has significant potential as a candidate for an attenuated live vaccine.

Another critical requirement for developing an attenuated live vaccine is its ability to elicit both humoral and cellular immune responses in the host (Lin et al., 2017). A robust humoral immune response, particularly the secretion of secretory IgA (SIgA) in the intestinal mucosa, is essential for combating *Salmonella* infections (Roesler et al., 2006). Furthermore, as *Salmonella* is a facultative intracellular pathogen, cellular immune responses are crucial for effective host defense (Nandre et al., 2011). In this study, indirect ELISA revealed that Δ*mcpC* induced strong specific humoral and mucosal immune responses. Given that this pathogen primarily resides in the intestinal tract, IgA secreted into the intestinal lumen is likely to play a key role in protective immunity (Nandre et al., 2011). Our results also showed that Δ*mcpC* could induce a specific cellular immune response, and up-regulate the expression of IL-6, *IL-10*, and *IFN-γ*. IL-6 is pro-inflammatory cytokines that coordinate inflammatory and host defense responses (Huang and Sheng, 2010). *IL-10*, predominantly produced by Th2 cells, is a multifunctional anti-inflammatory cytokine associated with immune regulation, defense, and infection (Yuan et al., 2022). *IFN-γ*, produced by activated T cells and natural killer (NK) cells, plays a vital role in host defense against intracellular pathogens such as *Salmonella* Typhimurium (Benbernou and Nauciel, 1994). Immune protection rate is a key indicator of vaccine efficacy, reflecting the vaccine’s ability to protect against the target pathogen post-immunization. Oral immunization with Δ*mcpC* achieved a relative protection rate of 100%, demonstrating its strong protective efficacy in mice.

In summary, the *mcpC* gene is involved in multiple biological processes in *SE*, and its deletion significantly attenuates bacterial virulence in mice. The Δ*mcpC* strain was shown to induce robust immune responses and provide excellent immune protection in mice. These findings suggest that the Δ*mcpC* strain is a promising candidate for an attenuated live vaccine against *SE*, laying a preliminary foundation for the development of genetically engineered vaccines. Considering the broad host range of *SE*, future studies will validate its protective efficacy and safety in poultry and other animal models.

## Data Availability

The datasets presented in this study can be found in online repositories. The names of the repository/repositories and accession number(s) can be found in the article/supplementary material.
